# Abnehmende Morbidität und Letalität bei Kindern nach Behandlung einer akuten lymphoblastischen Leukämie: ein Bericht der Childhood Cancer Survivor Study

**DOI:** 10.1007/s00066-020-01725-6

**Published:** 2020-12-11

**Authors:** Martin G. Sauer

**Affiliations:** Department of Pediatric Hematology and Oncology, Medizinische Hochschule Hannover, OE 6780, Carl-Neuberg-Straße 1, 30625 Hannover, Deutschland

## Fragestellung

Risikoadaptierte Therapieansätze bei der pädiatrischen akuten lymphoblastischen Leukämie (ALL) zielen darauf ab, das Überleben bei Hochrisikokonstellationen durch Therapieintensivierungen zu verbessern und bei Standardrisikopatienten durch weniger intensive Behandlungsschemata Spätfolgen zu reduzieren. Während Ersteres gut gelang, war bisher weniger klar, ob auch Letzteres erreicht wurde. Dies zu beantworten, war Gegenstand der hier zu besprechenden Arbeit.

## Methoden

Es handelt sich um eine retrospektive, registerbasierte Analyse über 6148 pädiatrische Patienten, die zwischen 1970 und 1999 wegen einer ALL behandelt wurden, und zwar entweder nach Protokollen der 70er-Jahre (70s, hier noch keine Risikostratifizierung) oder nach Behandlungsschemata der 80er- und 90er-Jahre. Hier wurde jeweils in Standardrisiko- und Hochrisikogruppen (80sSR, 80sHR, 90sSR und 90sHR) stratifiziert. Eine letzte Gruppe stellen überlebende Rezidivpatienten bzw. solche dar, die sich einer Knochenmarktransplantation (R/BMT) unterzogen. Verglichen wurden die Inzidenzen der Spätletalität (nicht leukämiebedingt), von Zweitmalignomen (SMN), chronischen Beschwerden und des neurokognitiven Verlaufs. Gesunde Geschwisterkinder und die US-amerikanische Normalbevölkerung dienten als Negativkontrollen.

## Ergebnisse

Die Gesamtletalität nach 20 Jahren aus allen Gruppen lag bei 6,6 % (95 %-CI 6,0–7,1). Verglichen mit den 70s lag die Spätletalität der 90sSR und 90sHR mit 0,2 bzw. 0,3 deutlich niedriger („rate ratio“ [95 %-CI] 0,1–0,7) und war vergleichbar mit der standardisierten Letalitätsrate der US-Bevölkerung. Im Vergleich zu den 70s war in der 90sSR-Gruppe eine geringere Rate an SMN zu verzeichnen („rate ratio“ [95 %-CI] 0,3 [0,1–0,6]), die sich von der der normalen US-Population nicht unterschied. Ebenfalls waren in der 90sSR-Gruppe weniger chronische Beschwerden, ein geringeres Ausmaß an Gedächtnisschwäche und weniger Einschränkungen bei der Bewältigung bestimmter Aufgaben zu beobachten.

## Schlussfolgerung der Autoren

Risikostratifizierte Behandlungsansätze der pädiatrischen ALL haben Spätnebenwirkungen deutlich reduziert und die Spätletalität sowie das Auftreten von Sekundärmalignomen in einen der Normalbevölkerung vergleichbaren Bereich gebracht. Patienten, die in Hochrisikogruppen fallen, weisen jedoch weiterhin eine deutlich erhöhte Rate an Spätfolgen auf, das Spektrum hat sich allerdings verschoben.

## Kommentar

Die Behandlungserfolge der pädiatrischen ALL gelten als eine der großen medizinischen Errungenschaften des 20. Jahrhunderts. Diese Erfolge wurden im Wesentlichen durch die Kombination einer intensiven chemotherapeutischen Kombinationstherapie mit einer Schädelbestrahlung erreicht. Letztere senkte das Risiko für ZNS-Rezidive wesentlich [1, 2]. Diese Therapieerfolge waren jedoch mit signifikanten Spätfolgen wie Zweitmalignomen, Schlaganfällen, Kardiomyopathien und Defiziten in der neurokognitiven Entwicklung assoziiert. Über die Jahre konnten verschiedene biologisch-klinische, immunologische, zytogenetische und molekulargenetische Charakteristika der ALL mit unterschiedlichen Krankheitsverläufen assoziiert werden und ermöglichten so eine Risikostratifizierung. In der Folge fokussierten die Therapieansätze auf eine Intensivierung der Therapie in den Hochrisikogruppen, während eine Therapiedeeskalation bei Standardrisiko eine Reduktion der Spätfolgen bei anhaltend gutem rezidivfreiem Überleben sichern sollte. Dabei konnten beispielsweise die Schädelbestrahlung weitgehend durch CNS-gerichtete Chemotherapeutika ersetzt (intensivierte intrathekale Chemotherapie, Dexamethason, Hochdosis-MTX) und die kumulativen Anthrazyklindosen reduziert werden. Während diese Anstrengungen die gute Leukämiekontrolle in den Standardrisikogruppen verschiedener nationaler Studiengruppen bewahrten [3, 4], waren die erhofften Auswirkungen auf die Spätnebenwirkungen statistisch weniger leicht zu fassen. Nach unserem Kenntnisstand ist die vorliegende Arbeit die erste, die statistisch signifikant zeigen konnte, dass moderne Standardrisikotherapien der pädiatrischen ALL kein erhöhtes Letalitätsrisiko oder Zweitmalignomrisiko im Vergleich zur Normalpopulation mehr haben. Des Weiteren wurde das Risiko schwerer chronischer Folgeerkrankungen, wie der Entwicklung von Kardiomyopathien, Schlaganfällen und neurokognitiven Defiziten, signifikant gesenkt. Nichtsdestotrotz macht uns diese Arbeit deutlich, dass auch heute noch Kinder, die mit modernen Behandlungsschemata (90s) in Hochrisikogruppen behandelt wurden, zwar eine gesenkte Spätletalität, aber weiterhin statistisch ein signifikant erhöhtes Risiko für Zweitmalignome und chronische Folgeschäden haben. Interessanterweise scheint sich hier das Spektrum weg von Kardiomyopathien und neurokognitiven Defiziten hin zu Osteonekrosen und Diabetes mellitus mit entsprechenden Konsequenzen zu verschieben.

### Fazit

Durch die Risikostratifizierung von Kindern und Jugendlichen mit ALL können wir mittlerweile nicht nur die gute Leukämiekontrolle mit weniger intensiver Therapie bei Standardrisikopatienten erhalten, sondern auch die Spätletalität und -morbidität signifikant senken. Die Möglichkeit, die Schädelbestrahlung durch chemische und biologische ZNS-gerichtete Therapieverfahren zu ersetzen, hatte dabei einen bedeutenden Anteil. Bleibt die hypothetische Frage, was von einer putativen Gruppe 2020s zu erwarten wäre. Bestanden bis in die späten 90er-Jahre die Therapieschemata aus einer Kombination prinzipiell ungerichteter Therapieelemente, ermöglicht uns ein zunehmendes Verständnis biologischer und molekularer Zusammenhänge in der Leukämogenese die Entwicklung zielgerichteter und damit zunehmend spezifischer Therapeutika. Dieses wird die Behandlungsstrategien von Hochrisikopatienten grundlegend verändern und damit wahrscheinlich eine deutliche Reduktion der Spätfolgen auch für diese Patienten ermöglichen [5, 6].

*Martin Sauer, Hannover*
